# Iterative Reconstruction of Transcriptional Regulatory Networks: An Algorithmic Approach

**DOI:** 10.1371/journal.pcbi.0020052

**Published:** 2006-05-19

**Authors:** Christian L Barrett, Bernhard O Palsson

**Affiliations:** Bioengineering Department, University of California San Diego, La Jolla, California, United States of America; Boston University, United States of America

## Abstract

The number of complete, publicly available genome sequences is now greater than 200, and this number is expected to rapidly grow in the near future as metagenomic and environmental sequencing efforts escalate and the cost of sequencing drops. In order to make use of this data for understanding particular organisms and for discerning general principles about how organisms function, it will be necessary to reconstruct their various biochemical reaction networks. Principal among these will be transcriptional regulatory networks. Given the physical and logical complexity of these networks, the various sources of (often noisy) data that can be utilized for their elucidation, the monetary costs involved, and the huge number of potential experiments (~10^12^) that can be performed, experiment design algorithms will be necessary for synthesizing the various computational and experimental data to maximize the efficiency of regulatory network reconstruction. This paper presents an algorithm for experimental design to systematically and efficiently reconstruct transcriptional regulatory networks. It is meant to be applied iteratively in conjunction with an experimental laboratory component. The algorithm is presented here in the context of reconstructing transcriptional regulation for metabolism in *Escherichia coli,* and, through a retrospective analysis with previously performed experiments, we show that the produced experiment designs conform to how a human would design experiments. The algorithm is able to utilize probability estimates based on a wide range of computational and experimental sources to suggest experiments with the highest potential of discovering the greatest amount of new regulatory knowledge.

## Synopsis

In recent years, the exploration of life has been bolstered through the advent of whole genome sequencing. This new data source significantly enables the reconstruction of genome-scale metabolic networks. After a metabolic reconstruction, it will be necessary to discover the genetic control mechanisms that operate within an organism. Transcriptional regulatory network (TRN) reconstruction is costly both in terms of time and money, so it is critical that the reconstruction efforts be made as efficient as possible. Experiments must be designed so that the most new regulatory knowledge is discovered in each experiment. The huge number of possible experiments (~10^12^) and the vast amount of heterogeneous data available for designing experiments overwhelms the human ability to assimilate. The authors have developed an algorithm that utilizes a mathematical model of a reconstructed metabolic network integrated with a partially reconstructed TRN to identify the experiment designs with the highest potential of yielding the most new regulatory knowledge. The authors show that the produced experiment designs are similar to those a human expert would produce, and that the algorithm has a facility to incorporate any relevant data source to design such experiments.

## Introduction

As of January 2006, the TIGR Comprehensive Microbial Resource [1] contained 259 bacterial and 23 archaeal sequenced genomes, and the GOLD database [2] listed 987 ongoing prokaryotic sequencing efforts. The picture emerging from metagenomic [3] and environmental sequencing [4] efforts is that the number of sequenced genomes will surge in the near future. In order to further our understanding of these organisms, it will be necessary to reconstruct their various biochemical reaction networks. First will be metabolism, which is arguably the most basic function that a cell performs. After metabolic reconstruction [5], the second most feasible reconstruction will be that of transcriptional regulatory networks (TRNs). These regulatory reconstructions will require methods to systematically, comprehensively, and efficiently reconstruct TRNs for which little data exist. Initial work [6–12] on systematic TRN reconstruction has been performed. These pioneering efforts span the range from the theoretical to combined computational and experimental iterative methods, and they address many of the important issues in TRN reconstruction. No single method is available, though, that iterates between computational and experimental phases, utilizes a dynamic modeling framework, has a mechanism for incorporating probabilistic data derived from any source, and explores all of the ways that a network can be activated by different growth environments. All of these aspects are relevant to the “open question” of “whether automated experimental design can be useful in a large and poorly characterized biological system with noisy data” [7]. Since the functional state of a TRN is a direct consequence of its environment, an experiment design algorithm must comprehensively probe the TRN with different growth environments and must infer, based on whatever computational and experimental data that exist, which parts of the network will be most fruitful to target with experimental investigations. To be practical, the algorithm must suggest the most efficient series of experiments, for the potential number of experiments is vast.

**Figure 1:**
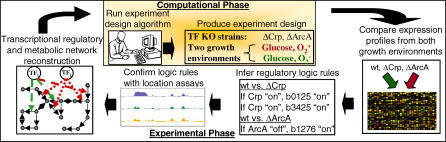
An Overview of the Combined Computational and Experimental Iterative Procedure The computational algorithm utilizes a dynamic simulation of the current integrated transcriptional regulatory and metabolic network reconstruction to design experiments. The new regulatory logic rules discovered by the experiments are then added to the reconstruction. TF_i_, transcription factor *i;* TF_j_, transcription factor *j*.

This paper presents an algorithm for systematically and efficiently reconstructing the topology and condition-dependent logic of TRNs. Practically, this means discovering new transcription factor (TF)–target gene regulatory connections, the (Boolean) logic of how TFs regulate target genes, and the environmental stimuli that modulate TF activity. The algorithm is presented here in the context of reconstructing transcriptional regulation for metabolism in Escherichia coli. The computational algorithm is intended to be applied iteratively, in conjunction with an experimental laboratory component that discovers direct TF–target gene interactions and the logic of the interactions (Figure 1). The computational component is based on dynamic growth simulations using regulated Flux Balance Analysis (rFBA), which is a constraints-based approach for bounding allowable steady-state metabolic network flux distributions, coupled with a Boolean logic formalism for transcriptional regulation. The laboratory component has been partially detailed elsewhere [13]. In summary, this experimental procedure employs single or double TF knockout (KO) strains to infer TF–target gene logic rules from a comparison between KO and wild-type gene expression profiles in two growth environments. This procedure is now augmented with TF binding assays to confirm direct TF interaction with target gene promoters [14–16]. In the context of this experimental protocol, an experiment design consists of a growth environment shift and a group of TFs (or “knockout group” [KG]) for which to create deletion strains. The purpose of the algorithm described herein is to produce such experiment designs, and to do so with the primary goal of maximizing the efficiency of the reconstruction process.

Efficient reconstruction means minimizing the number of experimental iterations. Efficiency gains are realized from fewer iterations in two key ways: time spent researching the next most informative experiment design (which could conceivably require weeks to months); and, laboratory supply and personnel costs required to perform each iteration. Minimizing the total number of iterations necessitates maximizing the number of new regulatory interactions discovered in each iteration. Given the experimental protocol described above, maximal rule discovery in each iteration depends on a simultaneous maximization and minimization. Assuming for a moment that one had a KG in mind for which one wanted to discover the TRN, it would be necessary to identify two growth environments that each maximally activate the regulatory connections for the identified TFs. Simultaneously, one would want to minimize the connections identically activated in both environments to minimize redundant discoveries.

This strategy is complicated by three facts. First, one would not have a complete TRN by which to judge whether the proposed growth environment shift–TF KG combination would be the one to yield the most new regulatory logic at the current stage of the reconstruction. Second, the best picture one could draw for the complete, real TRN would come from various data sources (e.g., literature, homology, expression profile based algorithms, location analysis, and TF binding site prediction algorithms) that are characterized by uncertainty and noise. Third, it would not be an optimal strategy to first choose a KG and then the best growth environment shift for that KG. Since the growth environment determines how a TRN is activated, and each of the possible KGs would be associated with different patterns of regulatory activity depending on the environment, it is the combination of growth environment shift and TF KG that determines the maximum yield of new regulatory logic.

The goal of designing a maximally efficient experimental strategy thus requires the resolution of three critical issues. First, how does one use criteria from various sources—with their attendant uncertainties, incompleteness, and noise—to infer as complete a picture as possible of the structure and logic of the TRN? Second, how does one utilize this incomplete picture to decide which growth environment shift–TF KG combination is most likely to yield the greatest amount of new regulatory knowledge? And third, how does one do this while steering away from previously discovered regulatory logic and towards new knowledge? After detailing the algorithm that addresses these issues in the next section, we describe how it has been applied and assessed in the Implementation section below.

## Results

### Algorithm

**Figure 2:**
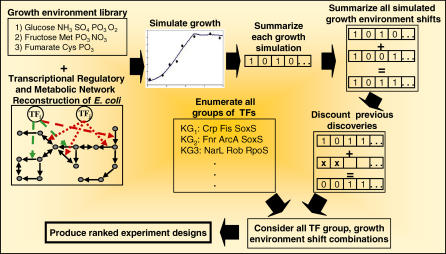
A Graphical Depiction of the Computational Algorithm The algorithm ultimately produces experiment designs ranked by their potential for producing the most new regulatory rules. TF_i_, transcription factor *i ;* TF_j_, transcription factor *j*.

The logic of the algorithm, depicted in Figure 2, is to simulate growth in a comprehensive suite of environments, and for each simulation, record the observed expression states of all genes and how the TRN was logically activated. This information is then used to identify the group of TFs (KG) that is most widely acting and most densely interacting, and the two growth environments (representing a growth environment shift) in which this occurs. The resulting combination of KG and growth environment shift is then considered the experiment design with the greatest potential for providing the most new regulatory rules. This presumption is based on the power law nature of TRNs [17,18], which implies that more highly connected TFs are more likely to be involved in undiscovered regulatory interactions. This methodology has the added advantage of (dis)confirming rules already in the model, but this becomes less of an issue as subsequent iterations utilize more speculative information in place of rules in the model as a basis for suggesting experiment designs. The algorithmic steps are detailed next.

#### Step 1: Simulate growth in an exhaustive set of environments and create corresponding activity profiles.

**Figure 3:**
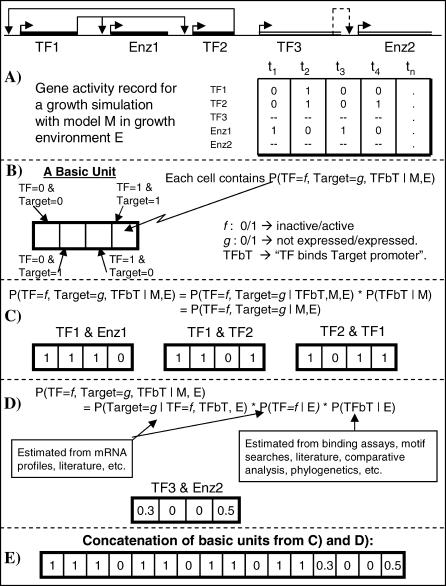
A Graphical Depiction of How an Activity Profile Is Created for a Growth Simulation The example model M in (A) contains genes for three transcription factors and two enzymes and shows the 0/1 (“off/on”) state of each gene in each time step t_n_ of a simulation for a defined growth environment E. The dashed line in the model indicates that a regulatory connection between the two genes is not explicitly modelled, but is suspected to exist. (B) shows how a “basic unit” is defined and shows the general formula for the parameterization of each cell. One basic unit exists for every known or suspected TF–target gene pair. As shown in (C), for such regulatory connections explicitly modelled, each cell of the basic unit gets either a “0” or a “1,” depending on whether its associated transcription factor and target gene were observed to be in the indicated 0/1 combination in any simulation time step. (D) illustrates how the inferred TF–target gene regulatory connections and logic derived from experimental and/or computational data are incorporated in a basic unit. In (E) the basic units from (C) and (D) are concatenated to form the activity profile for the simulation.

The procedure begins with a growth simulation using the E. coli model in each environment from a library of minimal media growth environments. The observed gene expression states and the logic of interaction between TFs and the genes they regulate are summarized in a single “activity profile” for each simulation (see Figure 3 and the first six sections of Materials and Methods for further detail).

#### Step 2: Identify all “legal” growth environment shifts.

Any two environments that define a shift must differ by only one component; otherwise, the inference of logic rules would be ambiguous because the true causative agent of a regulatory response would be ambiguous. Of the possible growth environment shifts, we identify the “legal” ones as those that differ by only one component.

#### Step 3: Create a shift activity profile for every legal growth shift.

A “shift activity profile” summarizes how the integrated transcriptional regulatory and metabolic (ITRAM) network is utilized in both environments of a growth environment shift. It is created by combining the two activity profiles of the two growth environments, as illustrated in Figure 2 and described in Materials and Methods. A shift activity profile is created for every shift identified in Step 2.

#### Step 4: Apply history mask to each shift activity profile.

To prevent the algorithm from re-suggesting previously implemented experiment design(s), a record is kept of those cells of the shift activity profiles that were used to suggest the experiments that were previously implemented. In this step, the history mask is applied to each shift activity profile generated in Step 3 (see Materials and Methods).

#### Step 5: Define all KGs of TFs.

Enumerate all combinations of TFs for KGs in a range of sizes (e.g., all combinations of two, three, four, and five TFs). (See Materials and Methods.)

#### Step 6: Quantify the interconnectedness of each KG in every shift.

The interconnectedness of a KG is the sum of regulatory connection weights between all pairs of TFs in a KG, where a regulatory connection is an instance in which one TF regulates the other, or the two TFs regulate a common (third) gene. The interconnectedness for a KG depends upon the particular growth environment. For each KG *k* defined in Step 5, calculate its interconnectedness (N_I_), N_I_(KG*_k_,* S*_ij_*), in each growth environment shift S*_ij_* from Step 2. (See Materials and Methods for further details.)

#### Step 7: Quantify the regulatory activity of each KG in every shift.

The regulatory activity of a KG is the sum of the TF–target gene regulatory interaction weights for all TFs in a KG, where a regulatory interaction is a probabilistic TF–target gene link. The regulatory activity for a KG will be growth environment dependent. For each KG *k* defined in Step 5, calculate its regulatory activity (N_R_), N_R_(KG*_k_,* S*_ij_*), in each growth environment shift S*_ij_* from Step 2. (See Materials and Methods for further details.)

#### Step 8: Identify the potentially most informative experiments.

With all growth environment shifts and all KGs enumerated, identify the growth shift–KG combination that maximizes first N_R_(KG*_k_,* S*_ij_*) and second N_I_(KG*_k_,* S*_ij_*). This ranking identifies the KG whose TFs' regulatory networks are both most widely influential and differently activated, and most densely interacting—and the two growth environments for which this is the case.

#### Step 9: Update history mask.

After an experiment design has been implemented in the experimental phase of the iterative cycle, a set of new logic rules will have been generated and old rules will have been confirmed. Some of these rules will be for TF–target gene interactions that were in the shift activity profile, whereas others will correspond to newly discovered regulatory interactions. For those rules in the former set, “mark” their corresponding cells in the history mask.

In practice, the algorithm does not produce just one experiment design. Since its purpose is to aid the experimentalist, it produces many designs in a structured format so that the experimentalist can factor in any additional criteria not considered by the algorithm. This aspect of the algorithm output is discussed further in the following Implementation section.

### Implementation

The issue of algorithm validation presents a difficult situation, for there is no complete TRN in existence for any organism. We were able, though, to perform a limited retrospective analysis using two experimental TRN reconstruction iterations performed before the development and completion of this algorithm. For these two experiments, human research and intuition were used to choose the growth environment shifts and TF KOs. The algorithm and its development were in no way influenced by the human-made choice of experiments for the first two iterations.

To date, only a limited amount of research has been reported towards the development of fictitious TRNs that would be complex, realistic, and logically consistent enough to test this algorithm. The logical consistency requirement is critical, for a “randomly-wired” TRN joined to a metabolic network would not be able to support simulated growth, and certainly not in a large number of growth environments. When such networks are successfully developed, they will be an additional test bed on which to evaluate the algorithm.

#### Human-produced experiment designs.

The two experimental reconstruction iterations previously performed are given in Table 1. The first [13] utilized an anaerobic fermentation to aerobic shift with glucose as the carbon source. Five single-deletion KO strains, plus one double KO, comprised the KG. The second experimental iteration pursued an anaerobic fermentation to nitrate shift with glucose as the carbon source. As this second iteration is still in progress, only one TF (narL) has been investigated. Each of these iterations can be assessed on the number of new regulatory rules that they discover, and in particular how many of these new rules derive from each TF KO strain.

**Table 1:**
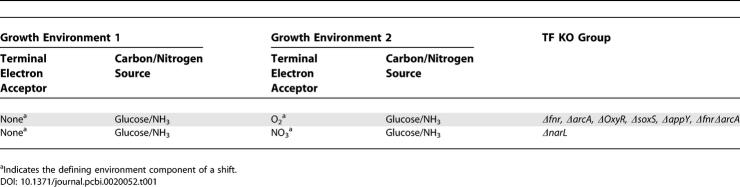
Human-Made Designs of Double Perturbation Experiments

Although the Δfnr and ΔarcA strains from the first iteration and the ΔnarL strain from the second iteration were found to be highly informative (producing roughly 130 new rules for *Δfnr* and *ΔarcA* and about 70 for *ΔnarL*), the *ΔsoxS* and *ΔappY* strains from the first iteration were found to be relatively uninformative; they produced 30 and four new regulatory rules, respectively. (The *ΔoxyR* strain gave an intermediate number of rules, but many were likely a result of an iron stress response. This result is being clarified, but it appears that the *ΔoxyR* strain was weakly informative.) These results suggest that more informative TF choices could probably have been made.

#### Comparison to algorithm-produced experiment designs.

For the comparison we implemented the algorithm using iMC1010^v1^, a genome-scale reconstruction of the integrated transcriptional regulatory and metabolic network for E. coli. For iMC1010^v1^, we constructed a library of 108,723 minimal media growth environments. By implementing Step 1 of the algorithm, we found that 15,580 of these were able to support growth. From these environments, we were able to form 21,121 legal growth environment shifts. Only published regulatory interactions (contained within iMC1010^v1^) were utilized, so all probability values in the activity profiles were unity. We considered KGs composed of between two and five TFs. For metabolic regulation in *E. coli,* for which iMC1010^v1^ currently includes 104 TFs, combinatorial calculation gives C(104,2) + C(104,3) + C(104,4) + C(104,5) = 96,748,106 unique KGs available for analysis. With 9.6 × 10^7^ KGs and 21,121 legal growth shifts for *E. coli,* this gives roughly 2 × 10^12^ potential experiment designs.

Table 2 shows the top ten ranked experiment designs that would have been suggested by the algorithm for the first iteration in Table 1. (The defining environment component of each shift is footnoted.) Table S1 gives database links for the TFs, and Table 2 is a low-detail output of the algorithm. In practice, for each design in Table 2, the algorithm produces many alternative, roughly equivalent growth environments that differ in their nitrogen, sulfur, phosphate, and (in the case of non-glucose growth environments) carbon sources. This high-detail output of alternative growth environments gives the experimentalist options and flexibility, for some suggested substrates may not be desirable to work with (e.g., substrate cost, poor utilization in batch culture, etc.)

**Table 2:**
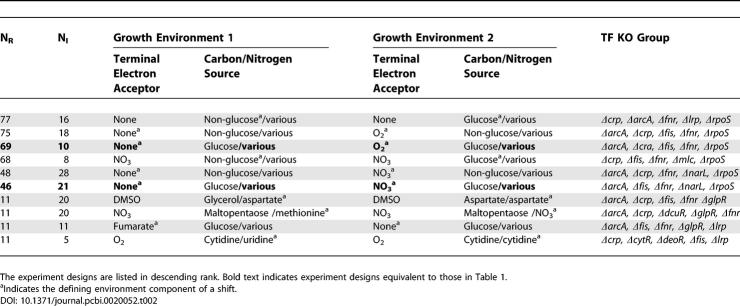
Computer-Generated Designs of Double Perturbation Experiments

Inspection of the growth environment shifts in Table 2 reveals that the first six shifts are between terminal electron acceptors (thus changing the respiratory conditions of the cell) or between glucose and non-glucose carbon sources. This behavior is in line with the human-conceived experiment designs from Table 1, which both utilized terminal electron acceptor shifts. This result is satisfying, for it supports our goal of developing an algorithm that designs experiments similarly to how a human expert would. Such a computational reasoning process will become especially advantageous when large amounts of speculative information (from high-throughput experimental and computational sources) are added to the design procedure, for such valuable information would overwhelm any person's reasoning ability. Explicitly, both human and algorithm sought two growth environments that activate the integrated transcriptional regulatory and metabolic (ITRAM) network in maximal, and maximally different, ways. And additionally, for maximal rule discovery, both targeted for KO those TFs most responsible for mediating the network activations.

The last four experiment designs are of a qualitatively different nature. These last four are mainly aimed at elucidating nitrogen-related regulation, and so do not probe regulation states that are as different as seen with electron acceptor and glucose shifts. They do this by shifting from a growth environment containing two components, where at least one can also function as a nitrogen source, to an environment containing just one component that functions as both the carbon and nitrogen source. These experiments are expected to reveal less, and less globally acting, regulation—as implied by the lower N_R_ values. The ranking of experiment designs in Table 2 illustrates how the algorithm works to uncover more global regulation first and then focuses on fine-grained resolution through discovery of more local regulation.

The TFs suggested for the experiment designs in Table 2 conform to the reasoning of targeting for KO those TFs most responsible for the network activations. For example, in correspondence with the top six suggested growth environment shifts being carbon source or respiratory condition shifts, the suggested TFs are widely influential glucose and carbon metabolism related (crp, mlc, and cra) and respiratory-state specific (arcA, fnr, and narL). Both fis and lrp are included because they are generally widely acting and interact at promoters with some of the other more global TFs, namely crp and arcA, and to a lesser extent, mlc and narL. The remaining TF, rpoS, is primarily associated with stress response and transition to stationary phase, both of which elicit large gene expression program changes. The TF rpoS is known to have a large regulon and to be present at low levels during log phase [19,20]. Whether or not to investigate rpoS during log phase as suggested by the algorithm would likely attract expert evaluation more than any of the other suggested TFs. Given the wide-ranging influence of rpoS, an expert may decide that an investigation of the role of rpoS in log phase is worthwhile, especially since a clearer understanding of its regulatory targets in log phase may clarify its function in stress and stationary conditions. Such an expert evaluation step is the setting for which the algorithm was designed.

Both of the experiment designs of Table 1 do occur in Table 2 (indicated by boldface text), but they are not the top-ranked designs. The first design from Table 1 is the third-ranked design in Table 2, with a slightly different repertoire of TF KOs. The experiment designs from both human and the algorithm suggest the fnr and arcA TF KOs, but they suggest different additional sets of three. It is not currently known whether the algorithm-suggested group of three TFs would be more informative than those chosen by humans, but as discussed above, at least two of the three suggested by human were found to be relatively uninformative. The second design from Table 1 is ranked sixth in Table 2, and both human and algorithm suggest the same TF that has so far been investigated in that iteration.

Just as the algorithm was run to retrospectively suggest experiments for the first iteration, we used the updated reconstruction resulting from the completed first iteration to retrospectively suggest experiment designs for the second iteration. We do not show an associated table for these results, but report that they are very similar to those of Table 2 with the difference that the third-ranked design in Table 2 is ranked sixth. The reason that this design dropped in ranking is due to the history mask, and demonstrates the role of the history mask in steering the algorithm away from previously implemented experiments.

## Discussion

### Intuitive Interpretation of the Algorithm

The rationale for Step 8 of the algorithm is based on the power law nature of TRNs [17,18], which implies that the most highly connected TFs are the ones most likely to be involved in undiscovered regulatory interactions. As a secondary objective, the algorithm seeks the most densely interacting group of TFs so that the more complex regulation will be identified first. This ordering of objectives is used so that the algorithm will function well for both associative and dissociative scale-free networks, in which in the latter the most connected nodes are not connected to each other.

To grasp why the algorithm produces the experiment designs that it does and how it arrives at a rank ordering of their information potential, it is helpful to consider a result from previous work [21] directly related to the algorithm work reported here. This result is included here as Figure S1. This figure is the result of clustering the activity profiles computed in Step 1 of the algorithm. It shows distinct clusters of activity profiles whose relative spatial arrangement is due to the growth environments to which the contained activity profiles correspond. In particular, it was found that the most prominent discriminators between activity profiles were the terminal electron acceptor present in the growth environment and whether or not glucose or gluconate was the carbon source. A secondary level of clustering, seen as distinct but overlapping clusters, was found to be due to the nitrogen source.

The observed clustering in Figure S1 both explains the experiment design algorithm's results and illustrates how it arrives at its design ranking. As discussed earlier, the intent of the algorithm is (in part) to identify two growth environments that each maximally activate the TRN, and that do so in the most dissimilar manner. Activity profiles in the same clusters of Figure S1 represent similar activations of the TRN, whereas those in different clusters represent more dissimilar activations—and to a degree directly corresponding to their separation distance. Because the terminal electron acceptor and the presence of glucose in the growth environment were found to be the prime discriminators between clusters, they primarily determine the dissimilarity of the two environments' activity profiles.

### Obtaining the Initial Network

The demonstration of the algorithm with E. coli represents a special case because of the large body of scientific literature that exists for E. coli. For the algorithm presented here to be applicable to an organism, there must be some initial characterization of its TRN. Because the algorithm presented here has the capability to incorporate and utilize essentially any source of probabilistic data through the basic units that constitute the activity profiles, it can be flexibly applied in an organism-specific manner since certain types of data may be more readily or inexpensively attainable, depending upon the organism. To mention a few possibilities, initial characterization could be achieved through inference of TFs in raw genomic sequence [22], of TF–target regulatory connections from ab initio prediction [23], mRNA expression profile data [24–26], or genome-wide location analysis [16], of TF binding sites from comparative genomics [27,28], and of network logic and structure from Bayesian and Boolean network–based methods [29].

### Relation to an Established Framework for Systems Biology

Systems biology is characterized by the integration of heterogeneous high-throughput data into a mathematical model and the subsequent use of this model to gain understanding of the cellular systems(s) under study in a way that would not be possible or feasible otherwise. Moreover, this is done iteratively, whereby new understanding is used to design subsequent experiments. Such a framework has been established and demonstrated in a four-step iterative procedure [30,31], but as was highlighted, one of the most striking challenges arising from systems biology is the further development of such iterative procedures. The method presented herein represents progress on this front. In the established work, experiments are designed to distinguish between models that explain biological data equally well. Here, experiments are designed for maximal discovery of new system knowledge. Additionally, the procedure presented herein integrates heterogeneous data into a dynamic model, which is arguably the type of model that will be required [32,33] for the full realization of the promises of systems biology.

### Other Modeling Frameworks

The algorithm has a modular aspect due to its ability to accommodate alternative transcriptional regulatory modeling strategies or different dynamical modeling frameworks altogether. In order to implement such modifications, four adaptations to the algorithm would be required. First, the basic unit will need to be able to capture the logical relationship between each TF–target gene pair, and do so in such a manner that external probabilistic data can be added. Second, a history mask will be needed that is appropriate for the new basic unit design. Third, a function for defining and quantifying the regulatory activity, N_R_, of a KG will be required. And last, depending on the new approach, a function for defining and quantifying the regulatory connectedness, N_I_, of a KG will be needed.

### Conclusion

We have presented an algorithm for systematically reconstructing a TRN with efficiency and human expert–like reasoning as prime considerations. Efficiency is based on time and cost; time is minimized through the algorithm-based experiment design process that would likely take a human expert weeks to months, and cost is minimized through the minimization of the number of laboratory experiments that need to be performed. The algorithm operates by deciding, given the current state of knowledge embodied in the TRN reconstruction, which experiment design—consisting of a group of TF KO strains and a growth environment shift—is most likely to yield the greatest number of new regulatory logic rules. The designs are equally applicable to overexpression studies. The algorithm has the ability to base its decisions on any data source that can assign a probability to the direct interaction of a TF and its regulatory targets and/or to the logical nature of its interactions. This aspect of the algorithm is significant, for it overcomes the finiteness inherent in a model and synthesizes a potentially vast amount of experimental and computational data that would not be possible by a human. We performed a limited retrospective comparison involving two previously performed TRN reconstruction iterations and found that the experiment designs that would have been suggested by the algorithm closely match the experiment designs that were chosen by human experts. This result illustrated our second goal of developing an algorithm with human expert–like reasoning ability. We expect this ability, when coupled with large amounts of probabilistic experimental and computational data, will significantly augment the limited assimilation ability of human experimentalists. Given the increasing number of organisms whose genomes have been sequenced and whose gene complement characterization is ever improving, we expect that this algorithm or others like or based on it will be necessary to efficiently uncover their transcriptional regulatory systems.

## Materials and Methods

### The model.

The algorithm presented herein is demonstrated using the first available genome-scale reconstruction of the integrated transcriptional regulatory and metabolic network for E. coli (iMC1010^v1^) [13]. It accounts for a total of 1,010 open reading frames (ORFs), or about one-third of the functionally assigned ORFs. It is composed of 906 metabolic ORFs enabling 932 biochemical reactions (including transporters) among 625 metabolites, as well as 104 TFs regulating the expression of 479 of the 1,010 ORFs. The reconstructed TRN is implemented with a Boolean logic formalism, which allows one to compute the “off/on” expression status of genes based on the “inactive/active” state of particular TFs and/or the absence/presence of particular environmental constituents.

### Growth simulations.

Dynamic batch culture growth simulations [34,35] were performed using the model under the regulated flux balance analysis (rFBA) [35–40] framework. In this framework, each simulation is an iterative process of short time intervals. In each iteration, the off/on status of all genes and the concentrations of environmental components from the previous iteration are noted and are then used to evaluate the (Boolean) transcriptional regulatory logic for every gene. The result is an updated off/on status for every gene, and thus every reaction, in the model. Then, using a steady-state flux assumption, a biomass pseudo-reaction is maximized, which results in the setting of all reaction flux values in the metabolic network. Based on these flux values, the concentrations of environmental constituents and the biomass are updated. This update completes an iteration. For this work, an environment that results in a biomass doubling time of at most 12 h is considered to allow cellular growth. Simulations corresponded to log-phase growth for 50 min, with time steps in 5-min intervals.

### Library of growth environments.

Every growth simulation must occur in a defined nutritional environment. In this work, we simulated growth in all possible minimal media growth environments. In order to enumerate all such possible environments, we collected all environmental components that could have an effect in the model described above and categorized each one as a carbon, nitrogen, phosphate, sulfur, or electron acceptor source. Some components were placed in multiple categories (e.g., glucose 6-phosphate serves as both a carbon and a phosphate source). Additionally, each category contains “None.” All combinations consisting of one component from each category formed the library of minimal media.

### The basic unit.

The central element in the algorithm is the basic unit. The purpose of a basic unit is to summarize the observed logical relationship between a single TF–target gene pair during a particular growth simulation. Implicit in this summary is also the expression states of the two genes. A basic unit is created for every TF–target gene pair between which a regulatory interaction is known or suspected to occur. Each basic unit is composed of four cells, which correspond to the four possible (Boolean) combinations of TF and target gene states (i.e., TF “inactive”/target “not expressed,” TF “inactive”/target “expressed,” TF “active”/target “not expressed,” and TF “active”/target “expressed”). Each of these four cells contains a single numeric value, which is a probability value reflecting the degree to which it is believed that the physical and logical interactions actually occur in the organism in the given growth environment. Each probability value is computed as the joint probability of the TF activity state *f*, the target gene expression state *g*, and of the event of the TF binding to the target gene's promoter (TFbT), or

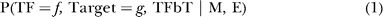
where M is the model and E is the growth environment.

### Parameterizing the basic unit.

Basic units are parameterized using data from two qualitatively different sources. These two approaches are discussed in the following two sections and are illustrated in Figure 3C and 3D.

### Parameterizing the basic unit using experimentally confirmed TF–target interactions.

For TF–target pairs whose direct interaction and logic of interaction have been experimentally confirmed (and so are explicitly accounted for in the model), the equation for parameterizing the cells of a basic unit is derived from Equation 1 and Bayes rule:

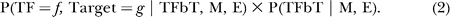



### Parameterizing the basic unit using speculative interaction information.

For regulatory connections between TF–target pairs that have not been experimentally confirmed but are suggested by data, computational and/or experimental data can be used to estimate the probability that (1) the TF represses/activates the target gene, (2) the TF is inactive/active in a particular growth environment, and/or (3) the TF binds to the promoter of the target. For integrating these varied types of data, Equation 2 is further expanded using Bayes rule to give

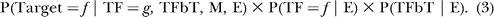



The first term is the probability that the TF is an activator (corresponding to the basic unit cells for TF/Target states 0/0 and 1/1) or is a repressor (corresponding to the basic unit cells for TF/Target states 0/1 and 1/0). The second term is the probability that the TF is inactive or active in the environment E. The third term is the probability that the TF binds the promoter for the target in E.

### Creating an activity profile for a growth simulation.

An “activity profile” summarizes the regulatory logic and gene expression states observed in a single growth simulation for the entire model. The procedure for creating an activity profile begins by recording the computed expression state of the genes in the model in every time step (see Figure 3). For those TF–target pairs whose regulatory connection is explicitly contained in the model, Equation 2 is used for placing values in the basic unit. Since we use experimentally confirmed data in the model, the second term in Equation 2—which can literally be interpreted as “the TF can bind the target promoter, given the model”—is taken to be unity. The first term is either 0 or 1 depending on whether the joint TF–target state combination was observed in any simulation time step. See Figure 3A and 3C for an illustration. Next, basic units for suspected TF–target interactions are parameterized using Equation 3 and any appropriate data (see Figure 3D). The final activity profile is then constructed by concatenating all of the basic units (see Figure 3E).

### Shift activity profile.

We used a “shift activity profile” to summarize the regulatory activity and gene expression states for a simulated shift between two growth environments. It is created from the two activity profiles corresponding to simulated growth in each of the two environments. The shift activity profile has the same dimensions as the activity profiles, and each of its cells has the larger of the two values observed in the corresponding cells of the two activity profiles. The larger value is chosen because it reflects the highest confidence of having observed the particular TF–target gene off/on state combination.

### History mask.

The algorithm presented herein is intended to be applied repeatedly and iteratively to systematically discover the TRN of an organism. To prevent the algorithm from repeatedly suggesting the same experiments, it is necessary to record which criteria were used to suggest any experiments performed in earlier iterations. As is explained in Steps 6–8 of the Algorithm section, these criteria consist of particular cells of the shift activity profiles. Thus, we record in a “history mask” those cells of the shift activity profile that were the criteria for choosing experiment designs that were actually implemented in previous iterations. The history mask has the same dimensions as a shift activity profile, and any cell whose corresponding logic has been confirmed by an experiment that was used to suggest a design is “marked.” The history mask is applied to each shift activity profile; those shift activity profile cells whose corresponding history mask cells are marked are overwritten with the value 0.0.

### KO groups.

One of the fundamental outputs of the algorithm is the identification of groups of TFs for which to create single-deletion KO strains. Such groups of TFs we term “knockout groups,” or KGs. A KG is characterized by the number of TFs it contains and the identity of the TFs. For a total of m TFs, there are C(m,n) unique KGs composed of n TFs.

### Quantifying the interconnectedness of a KG.

In regards to the TFs of a KG, we define two types of TF interconnections. In the first type, one TF directly regulates the other. In the second type, both TFs directly regulate a common (third) gene. To quantify the (total) interconnectedness of a KG *k,* it is first necessary to quantify the interconnectedness between every pair *p* of TFs in the KG. For this, we define I_total_(*p*| S*_ij_*) as the sum of the interconnection weights for all interconnections between the two TFs of *p* in growth environments *i* and *j*. For the first type of interconnection, the largest probability for such a physical interaction in both growth environments is used as the interconnection weight. In the second interconnection type, there will be a direct TF–promoter interaction probability for each TF in each of the two growth environments. The product of the two environment-specific probabilities is the probability that both TFs regulate the gene in that environment. The maximum of these two interaction probabilities is used as the interconnection weight. The interconnectedness of KG *k* in growth environment S*_ij_* is then computed as

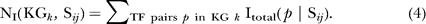



### Quantifying the regulatory activity of a KG.

We define the regulatory activity of a TF to be the probabilistically-weighted count of genes that it directly regulates. This number will be growth environment dependent. To quantify the regulatory activity of a TF *f,* we define R(*f* | S*_ij_*) as the sum over all connection weights between *f* and all of its inferred and/or known regulatory targets in both growth environments *i* and *j*. The connection weights are derived from the shift activity profiles in the following manner. First, all of the basic units for *f* in the shift activity profile for S*_ij_* are identified. For each basic unit that meets two conditions, its largest contained weight is added to R(*f* | S*_ij_*). The first condition states that the TF must be active in at least one of the environments *i* or *j,* because an experiment utilizing this growth environment shift with this TF KO strain would be uninformative otherwise. Specifically, this means that the last two cells of the basic unit, corresponding to TF “active”/target “not expressed” and TF “active”/target “expressed,” must both not be zero. The second condition states that the activity relationship between a TF and its target gene must have changed in the shift, otherwise the KO in the second environment may likely provide no new information. Specifically, the number of non-zero cells in the basic unit must be greater than one. The regulatory activity of KG *k* in growth environment S*_ij_* is then computed as

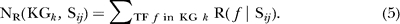



## Supporting Information

Figure S1 The Clusters of All Computation-Based Activity Profiles of iMC1010^v1^
The clusters are projected into three-dimensional space, allowing visualization of the “space” of transcriptional regulation and metabolic functional capabilities. The numbers in parentheses by each cluster in the key are the numbers of different activity profiles in the cluster. Comparison of the clusters shows that they can be distinguished by the available electron acceptor (indicated by the ellipses) and the carbon source, and to a lesser degree by the nitrogen source. The units of each axis are in bits, as given by the Hamming distance computed between computation-based activity profiles that are contained within the clusters.
(190 KB DOC)

Table S1 SwissProt Database Links for the Genes Mentioned in the Paper (32 KB DOC)

## Abbreviations

KG: knockout group
KO: knockout
NI: interconnectedness of a knockout group
NR: regulatory activity of a knockout group
ORF: open reading frame
TF: transcription factor
TRN: transcriptional regulatory network
