# Supplementary material for: Iterative Reconstruction of Transcriptional Regulatory Networks: An Algorithmic Approach
Source: PLoS Comput Biol. 2006 May 19;2(5):e52. doi: 10.1371/journal.pcbi.0020052 (PMC1463018; doi:10.1371/journal.pcbi.0020052)
Supplement: Supplementary file 1 [file pcbi.0020052.sg001.doc]

**Supplementary Figure 1:** The clusters of all computation-based activity profiles of iMC1010^v1^ projected into 3-dimensional space, allowing visualization of the “space” of transcriptional regulation and metabolic functional capabilities. The numbers in parentheses by each cluster in the key are the numbers of different activity profiles in the cluster. Comparison of the clusters shows that they can be distinguished by the available electron acceptor (indicated by the ellipses) and the carbon source, and to a lesser degree by the nitrogen source. The units of each axis are in bits, as given by the Hamming distance computed between computation-based activity profiles that are contained within the clusters.
