# Supplementary material for: Iterative Reconstruction of Transcriptional Regulatory Networks: An Algorithmic Approach
Source: PLoS Comput Biol. 2006 May 19;2(5):e52. doi: 10.1371/journal.pcbi.0020052 (PMC1463018; doi:10.1371/journal.pcbi.0020052)
Supplement: Supplementary file 2 [file pcbi.0020052.st001.doc]

Table S1: SwissProt database links for genes mentioned in the manuscript

| Gene name in manuscript | Swissprot primary accession number | Swissprot entry name |
| --- | --- | --- |
| fnr | P0A9E5 | FNR_ECOLI |
| arcA | P0A9Q1 | ARCA_ECOLI |
| oxyR | P0ACQ4 | OXYR_ECOLI |
| soxS | P0A9E2 | SOXS_ECOLI |
| appY | P05052 | APPY_ECOLI |
| narL | P0AF28 | NARL_ECOLI |
| crp | P0ACJ8 | CRP_ECOLI |
| lrp | P0ACJ0 | LRP_ECOLI |
| rpoS | P13445 | RPOS_ECOLI |
| fis | P0A6R3 | FIS_ECOLI |
| mlc | P50456 | MLC_ECOLI |
| dcuR | P0AD01 | DCUR_ECOLI |
| glpR | P0ACL0 | GLPR_ECOLI |
| cytR | P0ACN7 | CYTR_ECOLI |
| deoR | P0ACK5 | DEOR_ECOLI |
